# Closed-Loop Resonant Density Sensor Design Using Electromagnetic Excitation and Magnetic Detection

**DOI:** 10.3390/s25185740

**Published:** 2025-09-15

**Authors:** Jingyue Zhang, Lvjian Li, Jintao Wang, Xiang Liu, Xiaowen Su

**Affiliations:** 1 National Institute of Metrology, Beijing 100029, China; zhangjingyue@nim.ac.cn (J.Z.); llj_990302@163.com (L.L.); liuxiang@nim.ac.cn (X.L.);; 2 Shanghai Merchant Ship Design & Research Institute, Shanghai 201210, China

**Keywords:** density, structural design, resonant tube, electromagnetic induction, numerical simulation

## Abstract

With a hyperbolic U-tube as the resonant sensing element, the resonant density sensor adopts electromagnetic excitation and magnetoelectric detection for electromechanical transduction, enabling an integrated synergistic design. The resonance principle of the resonant density sensor and the electromechanical conversion method, using the electromagnetic induction principle, are analysed, and the theoretical model is investigated based on ANSYS Electronics 2022 and ANSYS Workbench 2022 R1 simulation software. In open-loop mode, the amplitude–frequency characteristics of the resonant network are measured, and the mechanical structure achieves a quality factor greater than 1000, as determined by the bandwidth method; In closed-loop mode, the measurement stability of the hyperbolic U-tube is periodically measured under various fluid loads, and the real-time ambient temperature is monitored. The sensitivity of the closed-loop system for density measurement is close to −0.1 Hz·kg^−1^·m^3^, and the absolute error between the density correction value and the standard value is within ±1 kg/m^3^.

## 1. Introduction

Microelectromechanical system (MEMS) [1] resonators have emerged as pivotal components in biosensing, mass detection, and core resonant elements of integrated circuits, leveraging their high sensitivity and miniaturisation capabilities [2]. Resonant sensors based on the inherent vibration characteristics of mechanical resonant-sensitive elements can use vibration frequency, phase, and amplitude as sensitive information parameters to achieve a variety of physical quantity measurements, with excellent repeatability, resolving power, and stability [3]. The basic structure of resonant sensors comprises a sensitive element, excitation unit, pick-up unit, and control circuit. The sensitive element, as the core of the resonant sensor, is directly linked to the physical quantity to be measured, and its vibration characteristics affect the performance of the resonant sensor [4]; the excitation unit and pick-up unit act as electromechanical conversion elements, and the coupling mode and degree between them affect the energy transfer efficiency; and the control circuit is used to maintain the interrelationships between the sensitive element and the excitation and pick-up units, and to provide the parameter output.

The selection of the excitation unit and pick-up unit and the coordination with the resonance-sensitive element are an important part of the structural design of the resonant sensor, which determines the relative position distribution between the three elements and the energy transfer loss of this structure [5]. The electromagnetic induction principle is applied in electromagnetic excitation and magnetoelectric detection, providing a non-contact way to achieve both energy supply to the resonant-sensitive element and vibration frequency acquisition from it, thus minimising possible adverse effects on the resonant-sensitive element [6].

Resonant density sensors exploit changes in the resonant frequency of a vibrating system to reflect variations in the density of the fluid within the sensitive element, thereby offering significant potential for high-precision density measurements [7]. In 1969, Austrian physicist Otto Kratky published a seminal paper that first introduced the concept of the resonant density sensor, laying the foundation for research in this field over the following decades [8]. His sensor employed a relatively rudimentary design, consisting of inlet and outlet tubes and a liquid reservoir sphere, and did not account for environmental influences on density measurements. In 1974, Patrick Picker and colleagues at the University of Sherbrooke in Canada extended the application of resonant densitometers to the field of process control. They used a stainless-steel V-shaped resonator as the sensing element and demonstrated that, for liquids with viscosities below 10 cp, viscosity had minimal impact on density measurements [9]. In 1980, Alfred P. Wenger of the Straumann Institute in Switzerland proposed a viscosity correction theory to address the issue of reduced accuracy of resonant densitometers in high-viscosity-liquid applications [10]. In 1984, Henry J. Albert and colleagues at the University of Delaware in the United States developed a single U-shaped resonant densitometer structure, enabling operation under extreme conditions of 700 K temperature and 40 MPa pressure [11]. In 1986, T. Retsina and colleagues at Imperial College London established a comprehensive mathematical model for vibrating-tube resonators, overcoming the prevalent reliance on empirical design approaches and providing a theoretical framework for sensor development [12]. In 2007, Edwin Krasser and colleagues at Graz University of Technology in Austria proposed a circuit architecture that integrates closed-loop self-excited oscillation with a digital phase-locked loop, thereby achieving the precise capture of the resonant frequency [13]. In 2019, Andreas Rechberger and colleagues at Anton Paar proposed the use of a pulse excitation method to determine the natural frequency and quality factor of the resonator, introducing a new paradigm into resonant density measurement [14]. More recently, in 2022, Olga Prokopová and colleagues focused on vibrating-tube densitometers operating at atmospheric pressure, employing single U-shaped glass resonators, and proposed an improved temperature calibration method along with an uncertainty evaluation strategy [15].

However, research on resonant density sensors based on electromagnetic excitation and magnetoelectric detection remains limited, with a lack of systematic theoretical analysis and experimental validation. In particular, the coupling characteristics between the mechanical structure and closed-loop circuitry have not been sufficiently explored, which restricts performance optimisation and hinders broader practical applications. Specifically, (1) early studies primarily focused on conceptual proposals and feasibility demonstrations, without providing systematic analyses of sensor performance [8]; (2) although some works introduced viscosity correction, temperature compensation, or circuit improvement strategies, their performance characterisation largely remained qualitative or only partially quantitative. For example, precision was vaguely described as “ppm-level accuracy” or as “approximately 0.1% within the ranges of ρ×η = 0.2 g/cm^3^ × 500 cp or 0.5 g/cm^3^ × 200 cp,” where ρ denotes density and η denotes dynamic viscosity, while comprehensive evaluations of sensitivity, resolution, and measurement uncertainty were lacking [9,10,11]; and (3) more recently, a pulsed excitation method was reported to achieve a quality factor of 2000 and a standard deviation of oscillation period measurements below 0.5 × 10^−9^ s. Nevertheless, its complex principle makes it more suitable for benchtop density meters, rendering it less applicable to portable devices, and further research is still required before practical deployment can be realised [14].

To address these issues, this study utilises a hyperbolic U-shaped resonant tube as the sensitive element, combined with a closed-loop self-excited oscillation circuit, to design and realise a portable resonant density sensor suitable for the high-precision measurement of small-volume liquids. Through the systematic analysis of the amplitude–frequency characteristics of the resonant network in open-loop mode, the quality factor of the mechanical structure was obtained. Furthermore, under closed-loop operation, the sensor’s measurement stability and density sensitivity were validated across four different fluids. The results not only enrich the theoretical framework of electromagnetic excitation and magnetoelectric detection resonant density sensors, but also provide essential theoretical support and technical guidance for their application in fluid density measurement.

## 2. Theoretical Analysis

### 2.1. Measuring Principle

The spring–mass system is employed as the mechanical model to describe the oscillatory behaviour of the vibrating tube density sensor [16], and this oscillation can be expressed by the following differential equation:

(1) $$\frac{d^{2}y}{dt^{2}}+\frac{b}{m}\frac{dy}{dt}+\frac{K}{m}y=\frac{F_{0}}{m}\cos(\omega t)$$

where *y* is the displacement from equilibrium; *b* is the damping coefficient; *K* is the spring constant; *F*_0_ is the amplitude of external periodic force; *ω* is the excitation frequency; *m* is mass; and *t* is time.

For the sensor’s mechanical structure, external force originates from the excitation unit, while damping arises from friction between the tube and air/fluid/pick-up unit [17]. The steady-state solution is

(2) $$y(t)=A_{0}\cos(\omega t-\varphi )$$

where *A*_0_ is the oscillation amplitude and *φ* is the phase difference between force and oscillation.

In this study, the electromagnetic force produced by the excitation coil serves as the external driving force. According to the local action principle of magnetic fields, magnetic energy is transferred through the presence and variation of the field in localised regions rather than via action at a distance [18]. Applying the virtual displacement principle, if the electromagnetic force vector *F*_0_ from the excitation coil induces air gap variation *dδ* with the proximal permanent magnet, the permanent magnet’s surface magnetic energy is expressed as

(3) $$W_{m}=\frac{1}{2}\int B\cdot H\cdot dV=\frac{B^{2}S}{2\mu_{0}}d\delta =F_{0}\cdot d\delta$$

where *B* is the magnetic flux density at the permanent magnet surface; *H* is the magnetic field strength at the permanent magnet surface; *V* is the variable volume of the air gap between the permanent magnet and resonator tube during vibration; *S* is the cross-sectional area of the hollow coil facing the permanent magnet; and *μ*_0_ is the vacuum permeability.

Based on the Ampere loop theorem and the magnetic circuit theorem, the magnetic induction strength *B* at the surface of the permanent magnet is the superposition of *B*_1_ (generated by the hollow coil) and *B*_2_ (from the static magnetic field of the permanent magnet):

(4) $$B_{1}=\frac{NI\cdot \mu_{0}}{l_{1}}$$

where *N* is number of turns in the coil; *I* is value of current passing through the coil; and *l*_1_ is integration path for the coil. Thus, we obtain

(5) $$F_{0}=\frac{{\left(B_{0}+B_{1}\right)}^{2}\cdot S}{2\mu_{0}}={\left(B_{0}+\frac{NI\cdot \mu_{0}}{l_{1}}\right)}^{2}\cdot \frac{S}{2\mu_{0}}$$

Neglecting minimal damping, resonant frequency equals natural frequency: $\omega =\omega_{0}=\sqrt{\frac{K}{m}}$. Given $\omega_{0}=\frac{2\pi }{T}$ (*T*: oscillation period), the density–period relationship is [19]

(6) $$\rho =\frac{K}{4\pi^{2}V}T^{2}-\frac{m_{0}}{V}=K_{2}T^{2}+K_{0}$$

where ρ is the sample solution density; *V* is internal volume of the vibrating tube (constant for a given tube); and *K*_2_ and *K*_0_ are calibration constants determined with two known-density liquids.

### 2.2. Structural Modelling

In this study, the resonant density sensor employs a hyperbolic U-tube as its sensitive element. Using the ANSYS Workbench modal analysis module [20], we performed modal simulation on this structure. By analysing the vibration characteristics of each modal order [21], symmetrical modes matching the first-order behaviour were selected as target modes.

**Figure 1:**
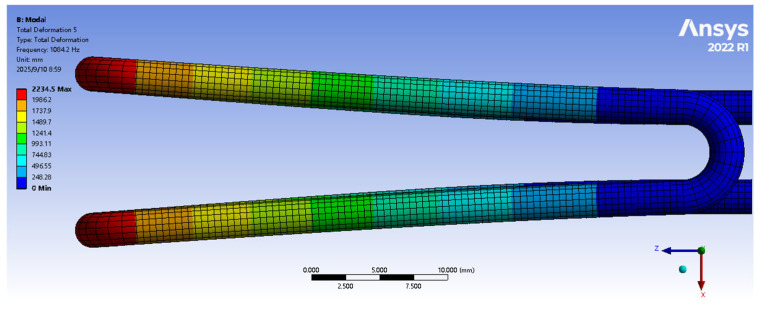
Maximum deformation cloud diagram of target modes in a hyperbolic U-tube resonant structure.

Figure 1 shows simulated target modes of a typical-sized hyperbolic U-tube resonator (top view). The cloud diagram depicts maximum deformation. To leverage the hyperbolic U-tube’s advantages, target modes must satisfy the following:

- Maximum deformation at the top of opposing U-tube arms;
- Symmetrical deformation (i.e., opposite directions at both ends).

Simulation results confirm that specific higher-order modes meet these requirements and can serve as target modes.

**Figure 2:**
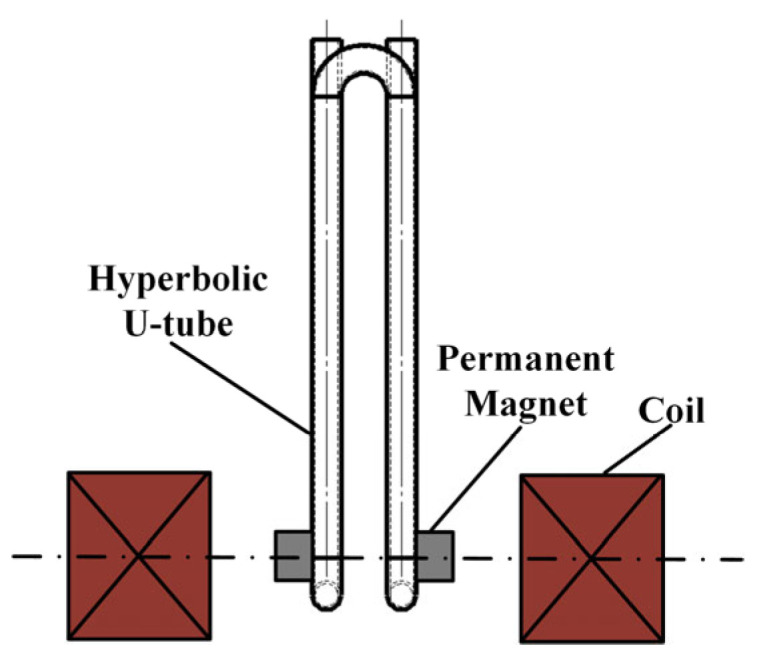
Single-end fixed-support double-curved U-tube mechanical structure.

This paper investigates a closed-loop system employing electromagnetic excitation and magnetoelectric detection. The resonator utilises non-magnetic borosilicate glass, which necessitates the symmetrical placement of bonded permanent magnets. Hollow coils and permanent magnets are coaxially arranged with an air gap maintained between them. Figure 2 illustrates the schematic structure and relative positioning.

**Figure 3:**
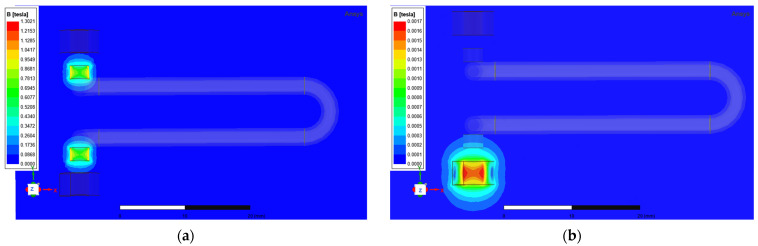
Magnetic induction intensity distribution contour plot. (**a**) Static magnetic field simulation of magnetic flux density contour plot; (**b**) magnetic flux density cloud diagram from transient eddy current simulation.

The hyperbolic U-tube top is selected as the excitation/detection location to maximise energy transfer efficiency. Magnetic field analysis of the model was conducted using ANSYS Electronics simulation software, and the magnetic induction intensity distribution contour plot is shown in Figure 3. Modelling parameters are based on the actual structure:

- Permanent magnet: 3 mm diameter × 2 mm height, coercivity > 835 kA/m, remanence ≈ 1.42 T;
- Copper coils: Upper coil (no excitation source) as pick-up coil; lower coil (ampere-turns is 6907 mA) as excitation coil.

Static magnetic field simulation analysis reveals a peak flux density of 1.3 T on the end surfaces of the cylindrical permanent magnets. The dominant permanent magnet field obscures the coil-generated flux, preventing clear observation of their interaction. Therefore, a further analysis of the energised coil was conducted in the eddy current simulation, where the excitation was set as stranded winding current excitation with parameters consistent with those used in the static magnetic field simulation. The magnetic induction intensity distribution contour plot from the eddy current simulation for this structural dimension indicates a maximum magnetic induction intensity of approximately 1.7 mT in the excitation coil, corroborating the overshadowing effect of the permanent magnet observed in the static magnetic field analysis.

## 3. Structural Design and Comparative Analysis

Resonant density sensors operate on the resonance principle, where fluid density changes shift the resonance frequency [22]. Excessively low frequencies increase susceptibility to external vibration noise, reducing the signal-to-noise ratio [23]; excessively high frequencies introduce parasitic circuit parameters and processing accuracy limitations, degrading the quality factor and measurement stability [24]. Therefore, optimal resonance frequency selection enhances density sensitivity, measurement resolution, and noise immunity while ensuring accuracy and reliability.

**Figure 4:**
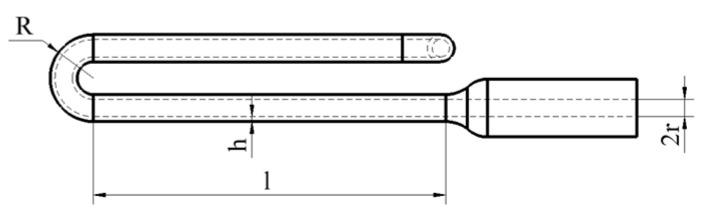
Side view of sensitive element.

This study employs a hyperbolic U-tube resonant sensor structure (side view: Figure 4). ANSYS Workbench modal simulations analyse how geometric parameters—inner radius *r*, wall thickness *h*, effective length *l*, and outer radius *R*—affect the resonance frequency of the U-tube.

**Figure 5:**
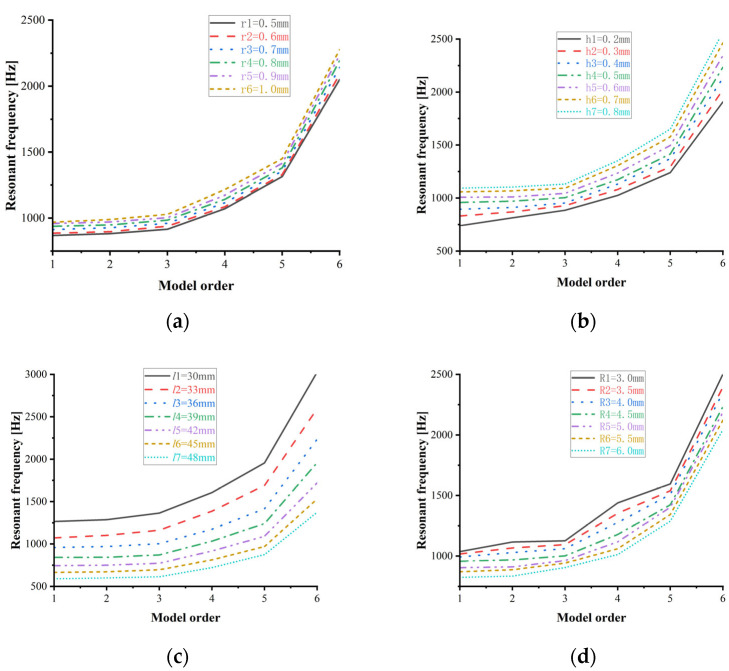
Influence of the structural parameters of the sensitive element on the resonance frequency plot. (**a**) Variation in resonance frequency with inner radius *r*; (**b**) variation in resonance frequency with wall thickness *h*; (**c**) variation in resonance frequency with effective length *l*; (**d**) variation in resonance frequency with outer radius *R*.

The typical resonator parameters of the hyperbolic U-tube are inner radius *r*_1_, wall thickness *h*_1_, effective length *l*_1_, and outer radius *R*_1_. Based on these parameters, we employ ANSYS finite element analysis to investigate how geometric parameters affect resonance frequency and select optimal values meeting design requirements. The parametric influence is shown in Figure 5.

Figure 5 shows the resonant frequencies of the first six modes for hyperbolic U-tubes as functions of inner radius (*r*), wall thickness (*h*), effective length (*l*), and outer radius (*R*). The dimensional parameters *r*1–*r*6, *h*1–*h*7, *l*1–*l*7, and *R*1–*R*7 represent incremental variations, with resonant frequencies of modes 1–6 exhibiting an overall increasing trend. Specifically,

- Modes 1–3 demonstrate smooth variations (<100 Hz);
- Modes 4–5 show distinct monotonic trends with reduced modal interference;
- Mode 6 exhibits a significant frequency increase.

Curve analysis reveals the following:

- Resonant frequencies increase with larger *r* or *h*;
- Resonant frequencies decrease with longer *l* or larger *R*;
- A physical mechanism.

Increased *r* or *h* enhances sectional stiffness and optimises mass distribution, strengthening the stiffness–frequency correlation. Conversely, increased *l* reduces axial stiffness and introduces additional mass, while larger *R* alters geometric configuration, weakening bend-region stiffness and extending the vibration path length. These effects collectively amplify inertial forces, reducing resonant frequencies. Rational dimensional design confines resonance frequencies within target operational ranges while maintaining structural stability.

## 4. Experimental Design and Analysis

**Figure 6:**
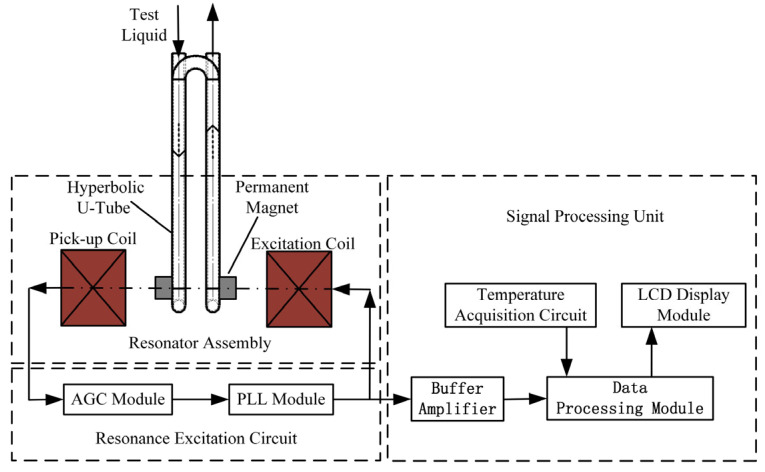
Block diagram of the overall sensor design solution.

Figure 6 shows the system architecture of the resonant liquid density sensor. The design integrates three functional modules: the resonator assembly, which governs core performance parameters; the resonance excitation circuit, which sustains mechanical oscillation; and the signal processing unit for outputting quantified measurements. Key performance indicators including quality factor (Q), density sensitivity, and measurement stability collectively define sensor efficacy [25]. The quantitative evaluation of these characteristics is provided through experimental validation in Section 3.

To ensure seamless integration between the mechanical resonant structure and its control circuitry for sustaining the continuous self-excitation of the sensing element in a stable first-order symmetrical mode, the closed-loop system must satisfy the Barkhausen criterion [26]:

(7) $$\left\{\begin{array}{l} \left|H(j\omega )\right|\cdot \left|G(j\omega )\right|\geq 1\\ \varphi [H(j\omega )]+\varphi [G(j\omega )]=2\pi \cdot n \end{array}\right.$$

where H(jω) denotes the frequency transfer function of the mechanical resonant structure; G(jω) represents the frequency transfer function of the resonant control circuitry; and n∈Z.

When the loop system, comprising the mechanical resonant structure and the control circuit, is powered on, weak noise signals are generated within the loop. These noise signals can be regarded as the superposition of numerous sinusoidal waves of different frequencies. After passing through the resonant sensing element, their amplitudes are attenuated and their phases are shifted according to the frequency response of the sensing element.

It is noteworthy that within the noise spectrum, there exists a specific frequency exactly matching the first-order symmetric resonant frequency of the sensing element. At this frequency, the amplitude attenuation of the noise signal is minimal, and the phase remains unchanged. When the gain of the control circuit is adjusted to precisely compensate for the attenuation at this frequency, while omitting phase adjustment, the noise signal at this frequency is amplified after passing through the closed-loop path, with its phase remaining constant.

For other frequency components, however, the amplitudes experience further attenuation and the phases undergo shifts. As the noise signals circulate repeatedly within the loop, the sinusoidal component that coincides with the first-order symmetric resonant frequency is gradually reinforced and eventually emerges from the noise, whereas all other frequency components continue to decay and remain as background noise. This mechanism, through positive feedback, continually amplifies the signal at the resonant frequency, ultimately sustaining the resonant state. Consequently, the resonant density sensor can stably output the first-order symmetric resonant frequency, which is directly related to the measured density.

### 4.1. Amplitude–Frequency Characteristic Testing Experiment

**Figure 7:**
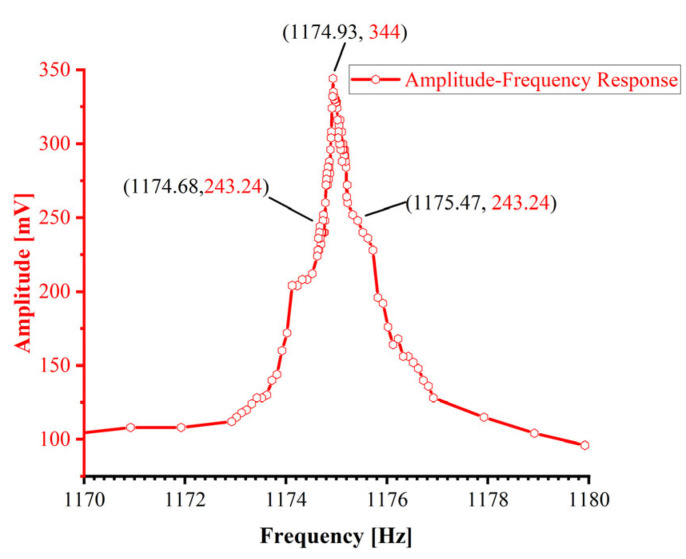
Amplitude–frequency characteristics of resonant networks in open-loop mode.

To eliminate interference from closed-loop control circuits during the dynamic characteristic evaluation of mechanical structures, open-loop frequency sweeps (1170–1180 Hz) characterise the amplitude–frequency response of the resonator assembly [27]. A 3 Vpp constant-amplitude AC excitation signal from a signal generator (SDG1022X, SIGLENT Technologies, Shenzhen, China) drives the sensitive mechanical element. The pick-up coil output is synchronously recorded by an oscilloscope (TBS1104, Tectronix, Beaverton, OR, USA) to acquire raw amplitude–frequency response data. Figure 7 depicts the amplitude–frequency response curve measured in air, showing a resonant frequency of 1174.93 Hz with an operational bandwidth of 1174.68–1175.47 Hz.

In sensor characterisation, the quality factor (*Q*) constitutes a critical parameter governing measurement precision, interference immunity, and operational stability. The quality factor *Q* is defined as 2π multiplied by the ratio of total stored energy to energy dissipated per oscillation cycle in a resonant system [28]. Practical *Q* values are conventionally acquired via indirect experimental methods. The bandwidth method represents one of the most established techniques for *Q*-factor quantification, derived from the frequency-to-bandwidth ratio at the half-power point of the sensitive element. This approach demonstrates robust reliability for systems where *Q* < 10^6^. The quality factor can be derived from the amplitude–frequency characteristics of resonant networks as follows:

(8) $$Q=\frac{f_{0}}{f_{H}-f_{L}}=\frac{1174.93}{1175.47-1174.68}=1487.25$$

In resonant sensors, the resonating element constitutes a weakly damped system, where the quality factor *Q* relates to the damping ratio *ξ* through the following expression [29]:

(9) $$\xi \approx \frac{1}{2Q}=\frac{1}{2\times 1487.25}=3.36\times 10^{-4}$$

### 4.2. Basic Calibration Experiments

**Figure 8:**
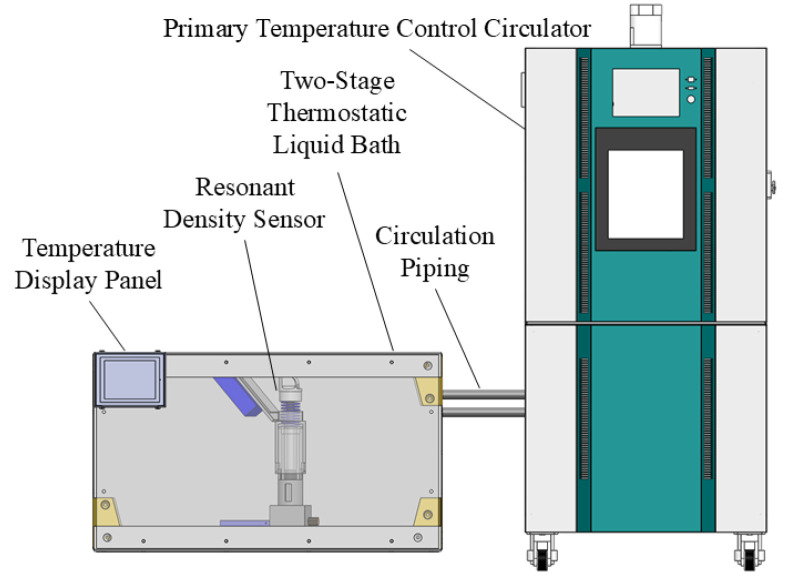
Schematic of the two-stage water bath temperature control setup.

The calibration experiment of the resonant density sensor needs to be conducted under a controlled temperature of 20 °C. Therefore, this study implements a two-stage water bath temperature control system, with the experimental setup shown in Figure 8.

As shown in Figure 8, the uncalibrated resonant density sensor is placed inside the two-stage constant-temperature water bath, and the real-time temperature inside the water bath is displayed on the temperature monitor. The temperature within the two-stage water bath is regulated in two ways: firstly, through a primary temperature control circulator connected via circulation piping, and secondly, by the water bath itself maintaining temperature stability. This two-stage control ensures stable temperature conditions within the water bath during the experiments. Moreover, both the basic calibration experiment and the temperature compensation experiment of the resonant density sensor are carried out using this two-stage water bath temperature control setup.

Resonant density sensors utilise mechanical resonance principles for high-precision density measurements, constituting an indirect metrological approach [30]. Density (ρ) serves as the target measurand, while resonance period (*T*) functions as the intermediate variable. Consequently, establishing the functional relationship between T and ρ is essential. As demonstrated in Section 1, the theoretical correlation between density and period follows Equation (6). To enhance fitting accuracy, a linear correction term is incorporated, yielding the final liquid density–resonance period relationship:

(10) $$\rho_{T}=K_{2}T^{2}+K_{1}T+K_{0}$$

where $\rho_{T}$ denotes the liquid density measured by the resonant sensor [kg/m^3^]; *K*_i_ represents the calibration coefficients of the resonant density sensor (*i* = 0, 1, 2); and *T* is the output resonance period of the densitometer [μs].

**Figure 9:**
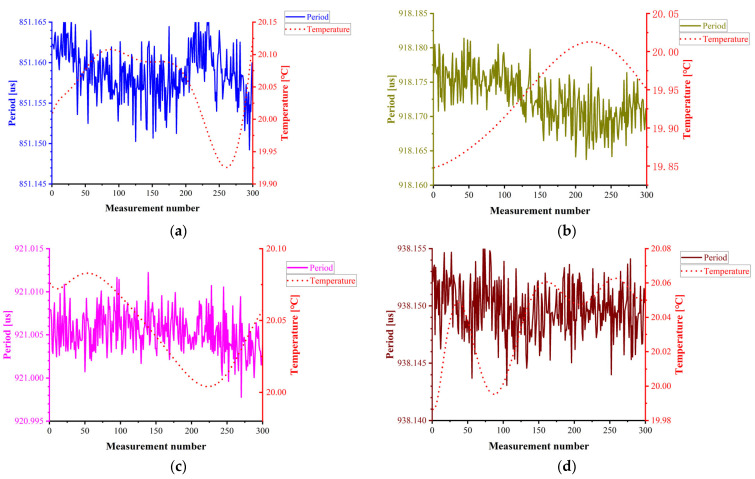
Experimental data for temperature calibration at 20 °C: (**a**) period variation of air at 20 °C; (**b**) period variation of Liquid 1 at 20 °C; (**c**) period variation of Liquid 2 at 20 °C; (**d**) period variation of Liquid 3 at 20 °C.

Calibration experiments are conducted under temperature-controlled conditions at 20 °C to eliminate thermal effects on resonant density sensor calibration. In this study, the period–density functional relationship is established by measuring the resonance period variations in air and three calibration liquids (denoted Air and Liquid 1–3) at 20 °C. The period fluctuation data for each fluid are presented in Figure 9.

The reference density values of the calibration liquids were measured using a DMA5000 benchtop densitometer (Anton Paar, Graz, Austria), achieving a measurement accuracy of 5 × 10^−3^ kg/m^3^ [31]. This precision satisfies calibration experimental requirements. Table 1 presents the period values and corresponding reference densities of all fluids at 20 °C.

The fundamental density equation for the resonant liquid density sensor is derived as follows:

(11) $$\rho_{T}=0.00896371T^{2}-4.5778T-2595.87$$

The measured period values are then substituted into the fundamental density Equation (11) to calculate the densities of each calibration fluid, as summarised in Table 1. The absolute error between the calculated and reference densities remains within ±0.50 kg/m^3^, with relative errors consistently below ±1%.

### 4.3. Temperature Compensation Experiments

In liquid density precision measurement and control, temperature variation exerts a dominant influence on density [32]. Temperature compensation experiments constitute a critical procedure to prevent ambient temperature from compromising measurement accuracy and stability. While Equation (10) provides the fundamental calibration formula for resonant liquid density sensors, it neglects thermal effects. The implementation of Equation (12) enables temperature compensation for density measurements acquired via resonant sensors, thereby enhancing measurement precision.

(12) $$\rho_{t}=\rho_{T}[1+K_{3}(t-20)]+K_{4}(t-20)$$

**Figure 10:**
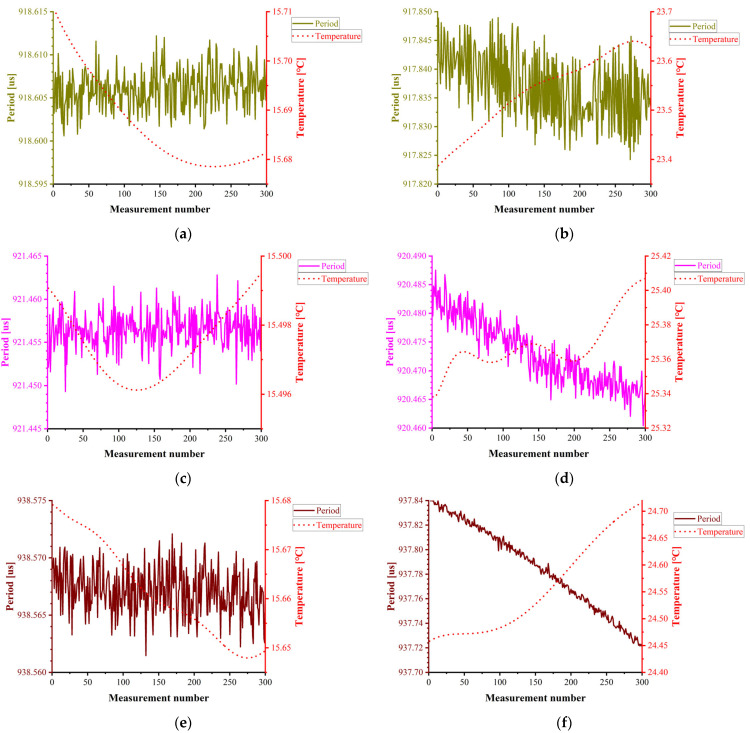
Temperature control experiment data at 15–25 °C. (**a**) Period variation of Liquid 1 at 15.7 °C; (**b**) period variation of Liquid 1 at 23.6 °C; (**c**) period variation of Liquid 2 at 15.5 °C; (**d**) period variation of Liquid 2 at 25.4 °C; (**e**) period variation of Liquid 3 at 15.6 °C; (**f**) period variation of Liquid 3 at 24.6 °C.

In this experiment, three calibration liquids (Liquids 1–3) underwent temperature-controlled testing from 15 °C to 25 °C. The temperature setpoints and corresponding period fluctuations are presented in Figure 10.

The DMA5000 benchtop densitometer (Anton Paar, Austria) employs advanced temperature-scanning technology, enabling the automatic temperature regulation of samples within preset ranges and real-time recording of corresponding density data. Leveraging this capability, the reference densities of three liquids at specified temperatures were measured.

Table 2 presents the resonance periods and corresponding reference densities of each fluid at temperatures ranging from 15 °C to 25 °C. The modified density equation for the resonant liquid density sensor is derived as follows:

(13) $$\rho_{t}=\rho_{T}[1+0.001741931(t-20)]-0.8620151(t-20)$$

The integrated model combining Equations (11) and (13) provides comprehensive density correction for the resonant liquid density sensor. Substituting measured period values into Equation (11) and subsequently applying Equation (13) yields calculated and corrected densities for each liquid (Table 2). The absolute error between corrected and reference densities remains within ±1.0 kg/m^3^, with relative errors below ±0.2%.

### 4.4. Density Measurement Sensitivity Analysis

Density measurement sensitivity constitutes a critical performance indicator for resonant density sensors, governing achievable measurement range and resolution. Higher sensitivity correlates with enhanced measurement resolution. Defined as the shift magnitude in resonant frequency per unit density change of the fluid within the resonance tube [33], this parameter ensures practical detectability: sufficient sensitivity amplifies frequency responses to minute density variations, while inadequate sensitivity may obscure density drifts due to inherent limitations in frequency measurement accuracy. The formal definition is provided in Equation (14).

(14) $$S_{i}=\frac{df_{i}}{d\rho_{l}}$$

Using predicted modal resonant frequencies in each medium as a reference, density measurement sensitivities were quantified across three transition ranges: (I) Air to Liquid 1, (II) Liquid 1 to Liquid 2, and (III) Liquid 2 to Liquid 3. This analysis utilised measured resonant frequency deviations and corresponding density differentials within each interval. The resulting sensitivities are compiled in Table 3.

As the liquid density increases, the density measurement sensitivity decreases. Theoretical analysis reveals two underlying mechanisms. First, increased liquid density augments the inertial load on the sensor’s resonant element (hyperbolic U-tube), elevating the equivalent mass of the resonant system. This mass gain compromises the operational efficiency of mechanical structures designed for non-extreme conditions. Second, higher-density liquids often exhibit increased viscosity, intensifying energy dissipation through boundary layer vibrations, consequently reducing frequency detection resolution.

## 5. Conclusions

This study focuses on liquid density measurement based on the resonance principle, utilising a hyperbolic U-shaped resonant tube with an electromechanical transduction method combining electromagnetic excitation and magnetoelectric detection to successfully establish a closed-loop measurement system. The system performance was comprehensively evaluated through metrics including the mechanical structure’s quality factor, density measurement sensitivity, and measurement stability. Experimental results demonstrate that the designed mechanical structure achieves a quality factor exceeding 1000, indicating excellent vibration energy retention. The density measurement sensitivity is approximately −0.1 Hz·kg^−1^·m^3^, which approaches that of some mature commercial products but still exhibits a noticeable gap, suggesting significant potential for further optimisation, particularly through adjusting the size and geometry of the hyperbolic U-shaped resonant tube. Within the temperature range of 15 to 25 °C, the absolute error between the density compensation values and standard references remains within ±1 kg/m^3^, confirming the good temperature stability of the sensor.

However, this study did not address viscosity compensation of the resonant density sensor, resulting in considerable measurement errors for high-viscosity fluids and limiting its applicability in complex fluid environments. Future work should focus on developing compensation algorithms for viscosity effects and investigating their impact on measurement accuracy to enhance the sensor’s versatility and practical utility.

## Figures and Tables

**Table 1:** Calibration of liquid resonance period and its density table.

Fluid	Air	Liquid 1	Liquid 2	Liquid 3
Period [μs]	851.12	918.17	921.00	938.11
Standard density [kg·m^−3^]	1.223	758.019	790.953	998.207
Calculation of density [kg·m^−3^]	1.231	757.663	791.363	998.166
Absolute error [kg·m^−3^]	0.008	−0.356	0.410	−0.041
Relative error	0.654%	−0.047%	0.052%	−0.004%

**Table 2:** Temperature-compensated liquid resonance period and its density table.

Liquid	Liquid 1		Liquid 2		Liquid 3	
Temperature [°C]	15.7	23.6	15.5	25.4	15.6	24.6
Period [μs]	918.60	917.83	921.46	920.47	938.57	937.77
Standard density [kg·m^−3^]	760.499	756.190	794.150	788.666	999.410	997.124
Calculation of density [kg·m^−3^]	762.774	753.624	796.854	785.040	1003.799	994.006
Corrected density [kg·m^−3^]	760.767	755.246	794.486	787.770	999.898	998.005
Absolute error [kg·m^−3^]	0.268	−0.944	0.336	−0.896	0.488	0.881
Relative error	0.035%	−0.125%	0.042%	−0.114%	0.049%	0.088%

**Table 3:** Density measurement sensitivity at 20 °C.

Fluid	Air	Liquid 1		Liquid 2		Liquid 3
Period [μs]	851.12	918.17		921.00		938.11
Frequency [Hz]	1174.92	1089.12		1085.78		1065.97
Calculation of density [kg·m^−3^]	1.231	757.663		791.363		998.166
Density measurement sensitivity [Hz·kg^−1^·m^3^]	−0.1134		−0.0991		−0.0958	

## Data Availability

Data are contained within the article.
